# Control of cortical oscillatory frequency by a closed-loop system

**DOI:** 10.1186/s12984-018-0470-z

**Published:** 2019-01-09

**Authors:** Mattia D’Andola, Massimiliano Giulioni, Vittorio Dante, Paolo Del Giudice, Maria V. Sanchez-Vives

**Affiliations:** 1 Systems Neuroscience, IDIBAPS, Rosselló 149-153, Barcelona, 08036 Spain; 2 Istituto Superiore di Sanità, Rome, Italy; 3 ICREA, Passeig de Lluís Companys, 23, Barcelona, 08010 Spain

**Keywords:** Stimulation, Cortex, In vitro, Real-time, Slow oscillations, Emergent properties, Direct current stimulation, Brain stimulation

## Abstract

**Background:**

We present a closed-loop system able to control the frequency of slow oscillations (SO) spontaneously generated by the cortical network in vitro. The frequency of SO can be controlled by direct current (DC) electric fields within a certain range. Here we set out to design a system that would be able to autonomously bring the emergent oscillatory activity to a target frequency determined by the experimenter.

**Methods:**

The cortical activity was recorded through an electrode and was analyzed online. Once a target frequency was set, the frequency of the slow oscillation was steered through the injection of DC of variable intensity that generated electric fields of proportional amplitudes in the brain slice. To achieve such closed-loop control, we designed a custom programmable stimulator ensuring low noise and accurate tuning over low current levels. For data recording and analysis, we relied on commercial acquisition and software tools.

**Results:**

The result is a flexible and reliable system that ensures control over SO frequency in vitro. The system guarantees artifact removal, minimal gaps in data acquisition and robustness in spite of slice heterogeneity.

**Conclusions:**

Our tool opens new possibilities for the investigation of dynamics of cortical slow oscillations—an activity pattern that is associated with cognitive processes such as memory consolidation, and that is altered in several neurological conditions—and also for potential applications of this technology.

## Background

The control of brain activity to restore function ideally requires the recording, or reading, of such activity, its online analysis and the correct stimulation/inhibition to achieve a target, which is a desired activity pattern. If this is done with periodicity, the system is defined as a closed loop. In order to gain control over the modulated parameters in electrophysiology, neuroscientists have employed closed-loop systems at different levels ranging from single-cell to network levels. At the single-cell level, notable examples are the voltage-clamp technique used in 1952 by Hodgkin and Huxley to design their model [1], or the dynamical-clamp technique introduced in 1993 by Sharp and colleagues to add in-silico conductances to the biological ones [2]. More recently, at the network level, different groups have demonstrated their ability to control the bursting frequency in in-vitro preparations [3, 4], and Jackson and colleagues were able to induce plasticity in the motor cortex during an in-vivo experiment thanks to an implantable closed-loop device [5]. Furthermore, a closed-loop system using alternating current (AC) stimulation of the human temporal cortex has been recently found to be useful for improving episodic memory [6] (for a review see [7]).

In the realm of clinical applications, even though some brain stimulation procedures, such as deep brain stimulation in Parkinson’s disease [8], have been well established for over two decades, the use of closed-loop systems associated with deep brain stimulation is recent and still experimental [9] despite their advantages [10]. In order to advance the design and use of closed-loop systems for the control of brain activity, in the current study we designed and tested a closed-loop system that controls the frequency of cortical slow oscillations (SO) by means of direct current (DC) stimulation of the cortical network, and we demonstrate that it is a flexible and reliable system.

SO are the dominant activity pattern of the cerebral cortex spontaneously emerging in various states, including slow-wave sleep, deep anesthesia, and in some brain areas after lesions (e.g., cortical islands). SO also emerge spontaneously in acute cortical slices in vitro in the absence of any pharmacological or electrical stimulation [11, 12]. The tendency for the cortical network to generate SO has prompted the suggestion that this activity is indeed the default activity pattern of the cortex [13] (reviewed in [14]). Furthermore, it is well established that SO during slow-wave sleep are associated with consolidation of information, and with enhanced memory and cognitive performance [15, 16] (reviewed in [17]). SO alternate at a frequency of ≤1 Hz between active and silent periods of neuronal activity [18], also referred to as Up and Down states, respectively. The neuronal firing of both excitatory and inhibitory neurons, which are linked by recurrent connections, results in reverberant or persistent activity during Up states. During Up states there is synchronization in beta (15–30 Hz) and gamma (30–90 Hz) frequencies, similar to what can be observed during wakefulness [12, 19]. These frequency bands during wakefulness have been associated with cognitive processes, short-term and working memory [20, 21], as well as with attention and arousal levels [22–25].

Interestingly, SO in in-vitro cortical slices [11, 12] can be modulated by DC stimulation. This is particularly relevant given that transcranial direct current stimulation (tDCS) has acquired a lot of attention in the past few years but, in spite of the extended use of tDCS, the network and cellular mechanisms underlying cortical DC stimulation are only partially known [26–28]. Application of DC electric fields parallel to the polarization direction of pyramidal neurons (i.e., perpendicular to the cortex layers) to act on the cortical activity modulates many network parameters characterizing SO, such as the frequency: the logarithm of the SO frequency varies linearly with the intensity and the polarity of the stimulation [28]. This effect is due to an elongation or shortening of the Down state duration due to hyperpolarizing or depolarizing fields, respectively, while Up state duration stays constant across stimulation levels.

Here we envisioned a closed-loop system that controls SO frequency in the cerebral cortex network in vitro as a proof of concept of the possibility to control this activity in vivo, eventually with therapeutic applications. This system is autonomous, able to stabilize the SO frequency, robust across time and flexible, as the intensity of the network response to the stimulation varies across slices [28]. In the following sections we present our closed-loop system as a proof of concept: it allows us to follow and to control the time evolution of the biological activity by controlling the injected current. Our closed-loop system is based on a commercial low-noise acquisition tool, a standard non-real-time PC, and a custom reprogrammable DC stimulator. An abstract of this article was previously published in a conference proceedings [29].

## Methods

The aim of this study was to design a flexible and reliable compact closed-loop system able to control the frequency of SO spontaneously generated by the cortical network in vitro. For this, we designed an experimental protocol consisting of two main parts: an open-loop part, for response characterization of the cortical network response; and a closed-loop part, where the biological activity was steered towards the desired SO frequency (see *Experimental protocol* below).

### Setup

**Fig. 1:**
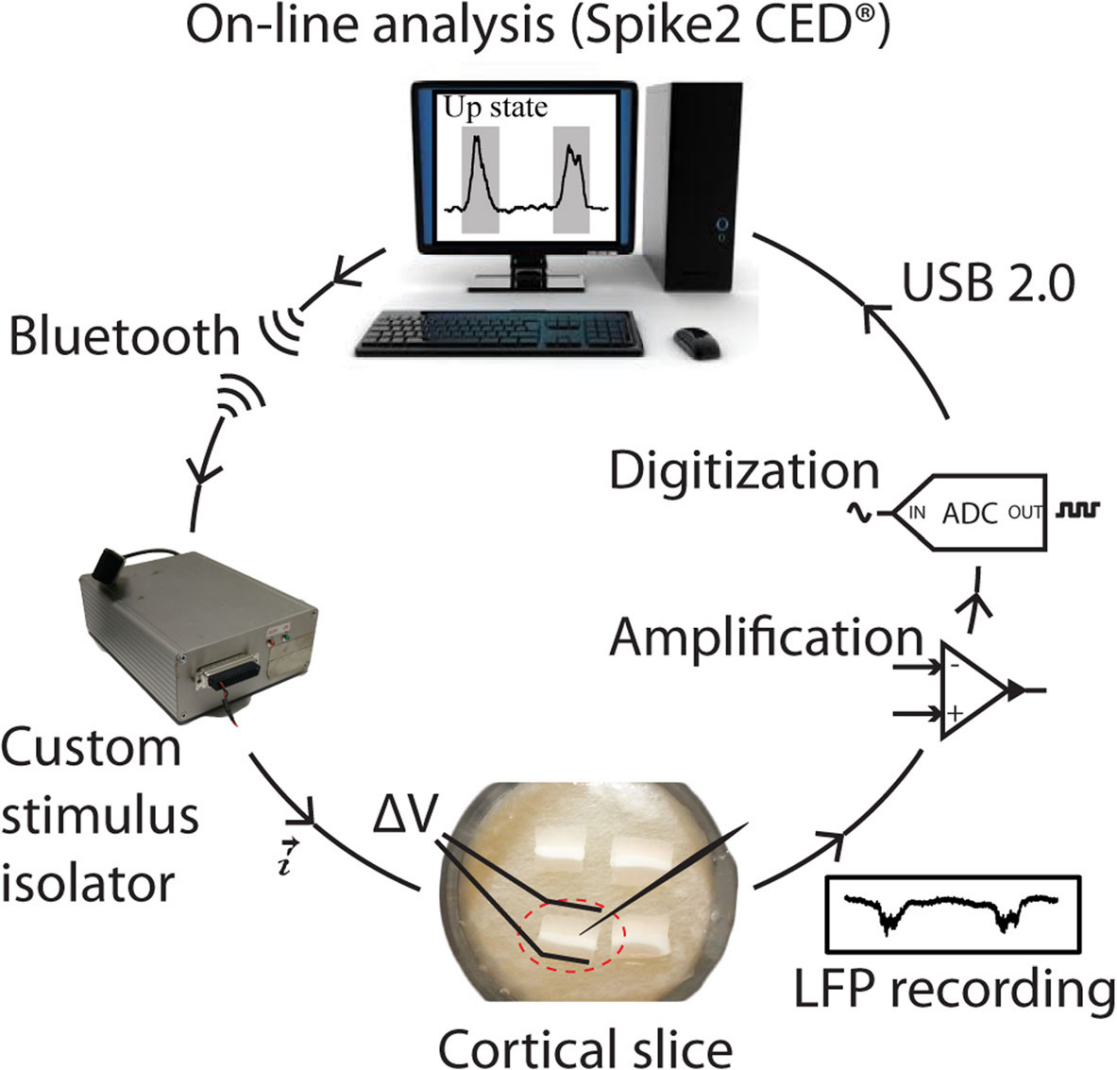
Closed-loop system. At the bottom of the figure is a photograph of four cortical slices in the recording chamber. In a counterclockwise direction, the loop proceeds from recording to stimulation. To the right, a sample of the local field potential (LFP) signal recorded from layer 5 is amplified, digitalized and sent to the PC via USB. At the top, the PC performs the online analysis for Up-state recognition and slow oscillation (SO) computation. To the left, the custom-made stimulator is controlled by the PC via Bluetooth. The stimulator injects DC current (*i*) into the slices by creating an adequate potential difference (ΔV) between the stimulation electrodes

Our setup was a loop composed of four main elements (Fig. 1): a cortical slice, a recording system, a PC for data analysis and a stimulator. Such a loop allowed us to continuously monitor the slow cortical oscillations, to compute their frequency (*v*) on-line and to adjust the stimulation current (*I*) to increase or decrease *v*.

A key feature of a closed-loop system is the ability to monitor, analyze and react in real-time to the biological behavior, “real-time” meaning that the time latencies of the loop should be much shorter than the time scales of the dynamics being studied [30–35]. In our case, latencies were not a tight constraint: up to hundreds of milliseconds were acceptable. We were interested in controlling the frequencies of slow oscillatory regimes and those frequencies rarely exceed 1 Hz. Hence we chose to record the slice activity through a commercial system that streamed the data on a Windows PC via USB and to run, on the same machine, the software for the continuous data analysis. The same software controlled the stimulator too. For us, three critical constraints arose that we had to take into account from the stimulation side, in which we used an adjustable DC stimulation current. Those constraints were: (1) the cortical network proved to react even to small (less than 10 μA) changes in current amplitude; (2) the nature of the stepwise stimulation did not allow for the use of standard techniques for artifact removal; and (3) we were interested in local field potential (LFP) signals (< 20 μV), thus a critical aspect was represented by the amount of noise introduced by the constantly active stimulator. Hence, to have fine control over the current amplitude, to avoid data loss after every stimulation update, and to maintain a low noise level, we designed a custom stimulator.

In the following sections we provide a detailed description of the various parts composing the setup.

### Slice preparation and experimental conditions

Ferrets were cared for and treated in accordance with Spanish regulatory laws (BOE-A-2013-6271), which comply with the European Union guidelines on protection of vertebrates used for experimentation (Directive 2010/63/EU of the European Parliament and of the Council of 22 September 2010). All experiments were approved by the Ethics Committee of the Hospital Clinic (Barcelona, Spain).

Slice preparation and solution composition are described in detail in Sanchez-Vives [36]. In brief: ferrets (4–6 months old, either sex) were anesthetized with sodium pentobarbital (40 mg/kg) and decapitated. Then the entire forebrain was rapidly removed and placed into oxygenated cold (4–10 °C) bathing medium and 400-μm-thick coronal slices from visual cortex (areas 17, 18 and 19) were obtained. Tissue viability was increased during preparation by modification of the sucrose-substitution technique [37]. The slices were then placed into an interface style recording chamber (Fine Science, Foster City, CA) where the slices were superfused for 1–2 h with a continuous solution flow (2–4 ml/min); the artificial cerebrospinal fluid (ACSF) contained (in mM): NaCl, 126; KCl, 2.5; MgSO_4_, 2; Na_2_HPO_4_, 1; CaCl_2_, 2; NaHCO_3_, 26; dextrose, 10; and was aerated with 95% O_2_, 5% CO_2_ to a final pH of 7.4. Then, a modified ACSF simulating ionic values in vivo (as described in [11, 36]), was used throughout the rest of the experiment, which had the same ionic composition except for different levels of the following (in mM): KCl, 4; MgSO_4_, 1; and CaCl_2_, 1. Bath temperature was maintained at 34–36 °C. Throughout the experiment the temperature was maintained at 34–36 °C.

### Electrophysiological recordings

The extracellular LFP was recorded from deep cortical layers with a 2–5 MΩ tungsten electrode (FHC, Bowdoinham, ME) and was amplified by 1000 by a Neurolog System (Digitimer, Letchworth, UK). The reference electrode, a Ag-AgCl wire, was placed in the bath. The amplified signal was digitized at 5 kHz by a CED instrument (Power1401 ADC/DAC, **C**ambridge **E**lectronic **D**esign, Cambridge, UK) and streamed to the PC.

### Slow oscillation identification and analysis

As mentioned in the introduction, SO are characterized by the alternation of active and silent periods, namely Up and Down states. During Up states, the increase in the spiking activity of single neurons increases multiunit activity (MUA) levels. A method to estimate MUA has already been described [28, 38]: in brief, the power spectrum of the population firing rate has Fourier components proportional to the firing rate itself [39]. Thus the components of the LFP between 0.2 kHz and 1.5 kHz can be seen as a linear transform of spiking activity [40], so that power changes in the Fourier components at those frequencies provide a reliable estimation of the population firing rate.

**Fig. 2:**
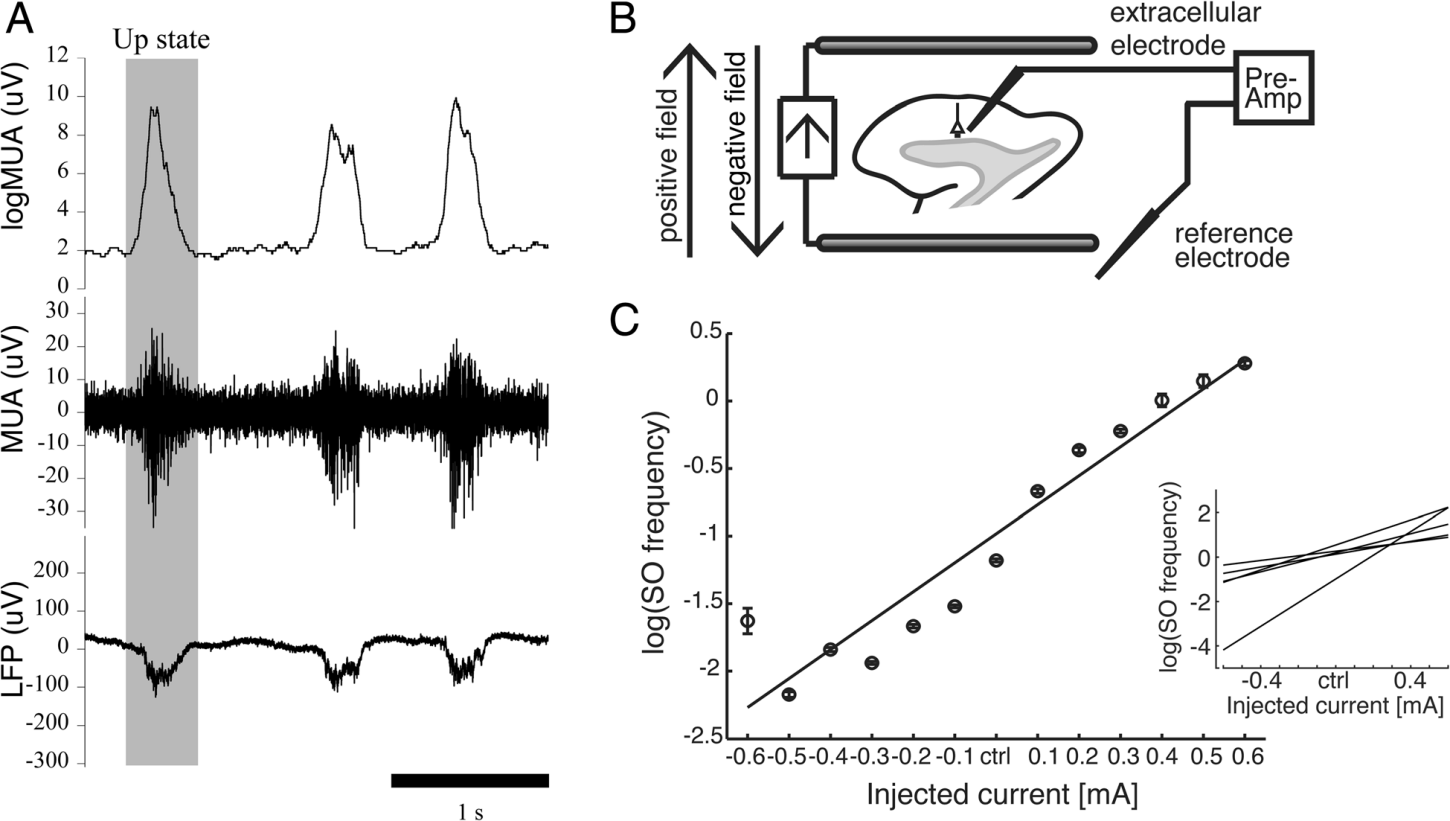
Sample recording traces, stimulation configuration and SO frequency behavior across injected DC currents. **a** Sample recording (3 s). Bottom row, raw LFP trace; middle and top rows, the MUA and MUA waveforms, respectively, obtained with online filtering of the LFP. Up states (gray shade) were automatically recognized through the use of amplitude and time thresholds in the MUA traces. **b** Scheme of electrode stimulation setup. Electric fields were directed parallel to the polarization direction of pyramidal neurons. **c** Typical distribution of SO frequency across injected currents for one of the recorded slices (mean ± SD). For each stimulation current (*x* axis), all the measured values of the slow oscillation frequency obtained during the stimulation interval at that specific intensity were averaged (58 values). The logarithm of the SO frequency monotonically increases with the applied DC currents. The linear fit is superimposed [28] (*p*-value = 3.9182 × 10^− 7^). The variability of the response to the stimulation across the five tested slices is showed in the inset by superimposing the linear fits for each slice, after normalization to the control condition (spontaneous activity). LFP, local field potential; MUA, multiunit activity; SO, slow oscillations

For an online estimation of MUA, we applied a DC-removal filter with a rectangular window of 10 ms (roughly corresponding to a high-pass filter with cut-off frequency at 100 Hz) and a second online moving-average filter with a rectangular window of 0.6 ms (roughly corresponding to a low pass filter with a cut-off frequency at 1700 Hz) to traces from LFP waveforms recorded during closed-loop stimulation (Fig. 2a). We then rectified the signal and applied another moving-average filter with a longer time-window of 80 ms to obtain MUA traces, similar to the ones obtained offline in [28, 38] (Fig. 2a). Upward and downward transitions — that is, from a Down state to an Up state, respectively, and vice versa — were detected by setting time and amplitude thresholds in the MUA traces. In order to obtain a better automatic recognition of the Up/Down cycles, the frequency cutoff of the bandpass filter for the MUA estimation was adjusted to [0.1 1.7] kHz on an empirical basis, with respect to the one reported in [40].

The detection procedure just described was performed by our Spike2 scripts which analyzed data online, detected the Up states and computed their onset frequency (i.e., the SO frequency) over a window of variable duration (see below). Up state onsets were identified as an increase in the MUA crossing a given threshold and lasting more than 200 ms. The threshold could be automatically adjusted by the software at a specific percentage of the amplitude of the MUA during Up states with respect to the baseline activity. At the same time it was also possible to adjust it manually at any moment of the recording. In the set of recordings that we will show, the threshold was either automatically set or manually adjusted throughout the experimental trials.

Every second the software compared the chosen target frequency $\Big(\overline{v}$) with the measured one (*v*) and increased or decreased the stimulation level by a certain current step (*I*_*step*_). The software acted as a proportional controller, such that the farther the signal was from the target the larger the current step was. When $\left|\overline{v}-v\right|\le 10\% of\ \overline{v}$, we considered the target had been reached and we did not modify the stimulation current. To avoid permanent damages to the slice we also enforced a maximum absolute value for the stimulation current of 650 μA. Current intensity ranges were chosen on the basis of previous observations [28] (see below).

To reduce false positives, the software detected and removed artifacts on the MUA channel before performing the analysis of the Up state onsets. Artifacts in the recordings derived from a sudden change of the voltage potential in the recording site caused by stimulus updates or by environmental interferences. Fast oscillations of the signal, creating a V-shaped peak of at least 30 μV height, were recognized by the system as artifacts. Apart from the real-time analysis, the software also ensured the execution of an entire experimental protocol involving different phases (see below).

### Stimulation

Stimulation was effected by a custom-made stimulator composed of an injection module, an Atmel microcontroller and a Bluetooth communication module. Wireless communication and battery powering were chosen primarily to get rid of ground loops that can arise in closed-loop systems that include multiple devices. In this way, and with additional measures including suited ground connections, we eliminated noise due to power lines, radio mobile signals and so on.

The microcontroller was in charge of managing wireless communication, DACs employed to set stimulation currents, and triggers for artifact removal.

The stimulator offers a command-line COM-based user interface for easy programming and provides four stimulation channels, independently settable. In the experiments reported in this work we used a single channel.

**Fig. 3:**
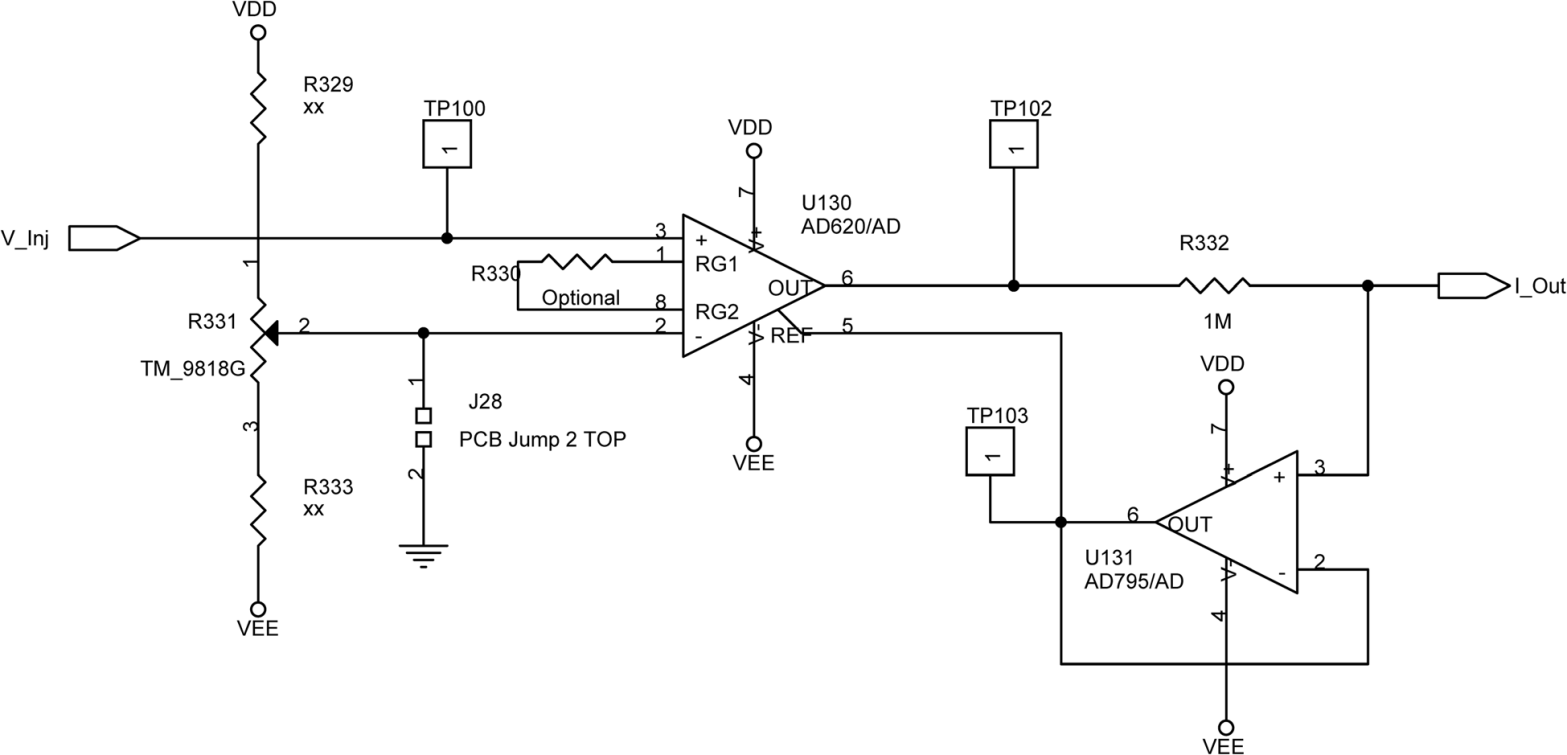
Diagram of the stimulator. Input voltage is applied at V_inj_, output current flows between I_Out_ and Gnd. The leftmost resistor branch ensures the current zero-point. AD62 0 AD is an instrumental amplifier; AD79 5 AD is an operational amplifier with very high input impedance. The configuration of the two amplifiers implements a feedback circuit based on the voltage across the R332 resistor, which dictates the V/I conversion factor

The current injector is basically a voltage-current converter and is illustrated in the diagram in Fig. 3. The instrumental amplifier and op-amp implement a feedback circuit that keep the voltage across the resistor R332 at the V__Inj_ value, which in turn determines the current I__Out_ to be injected.

Lab tests on the current injector confirmed linearity of the relationship between set and injected currents, up to clipping conditions that depend on the load impedance; such tests allowed us to easily assess whether the stimulation current is reliable by checking that, for the set current, the applied voltage is below the clipping value (about 8 V). All measurements were remarkably stable over time. For our injector, output noise can be safely estimated to be very small (3.3 μV peak-to-peak), based on the components data sheet and circuit schematics.

The circuit guarantees a linear relationship between the input voltage V__Inj_ and the injected current I__Out_ as long as the output voltage of the instrumental amplifier is below the clipping values VDD or VEE. We remark that the maximum injected current will obviously depend on the load downstream I__Out_. The injected current will be positive (negative) for positive (negative) V__Inj_. The availability of the test points TP102 and TP103 allowed us to check the V__Inj_ - I__Out_ ratio in situations where load increase and clipping conditions could be expected.

Every second the software instructed the stimulator to inject current into the cortical slice [26, 28, 41] through two parallel customized silver-chloride wires (1 mm diameter, 10 mm length) placed 5–8 mm apart (depending on the slice size), such that the electric field was oriented parallel to the apical-dendritic axis of cortical pyramidal cells (Fig. 2b). Positive fields are fields that depolarize the pyramidal neurons, oriented from the white matter to the cortical surface, while negative fields are oriented in the opposite direction, inducing hyperpolarization of pyramidal neurons. Homogeneity of the electric fields generated by the electrodes was tested in a separate study with a systematic voltage measure across the recording area.

Upon the arrival of a new command, the outputs were updated within a few microseconds and a digital trigger was activated on an optically decoupled dedicated channel. We connected this output to the CED instrument to mark current updates.

For the present work we used one single stimulation channel, we configured the stimulator to have reliable currents in the range − 1 mA to 1 mA settable with 100 nA accuracy for output load resistances in the order of few kOhms.

The intensity for the applied current was kept in the range [0 ± 0.65] mA. According to what has been previously observed [28, 41], in an interface chamber, where slices are bathed with the ACSF described before, electric fields were generated in the range 1–6 V/m. Current intensity and electric field intensity are usually linearly correlated in this range. This measure is not appreciably modified, neither by the presence of the slice nor by the recording electrodes [41]. As our custom stimulator injects current, we present here the effects on the cortical activity provoked by the stimulation by showing the amount of applied current instead of the obtained electric field intensity.

Some of the limitations of the present version of the stimulator will be overcome in an improved version (already designed) providing more channels, wider voltage range, optically decoupled input and output triggers, and embedded monitoring of V/I ratio.

### Experimental protocol

**Fig. 4:**
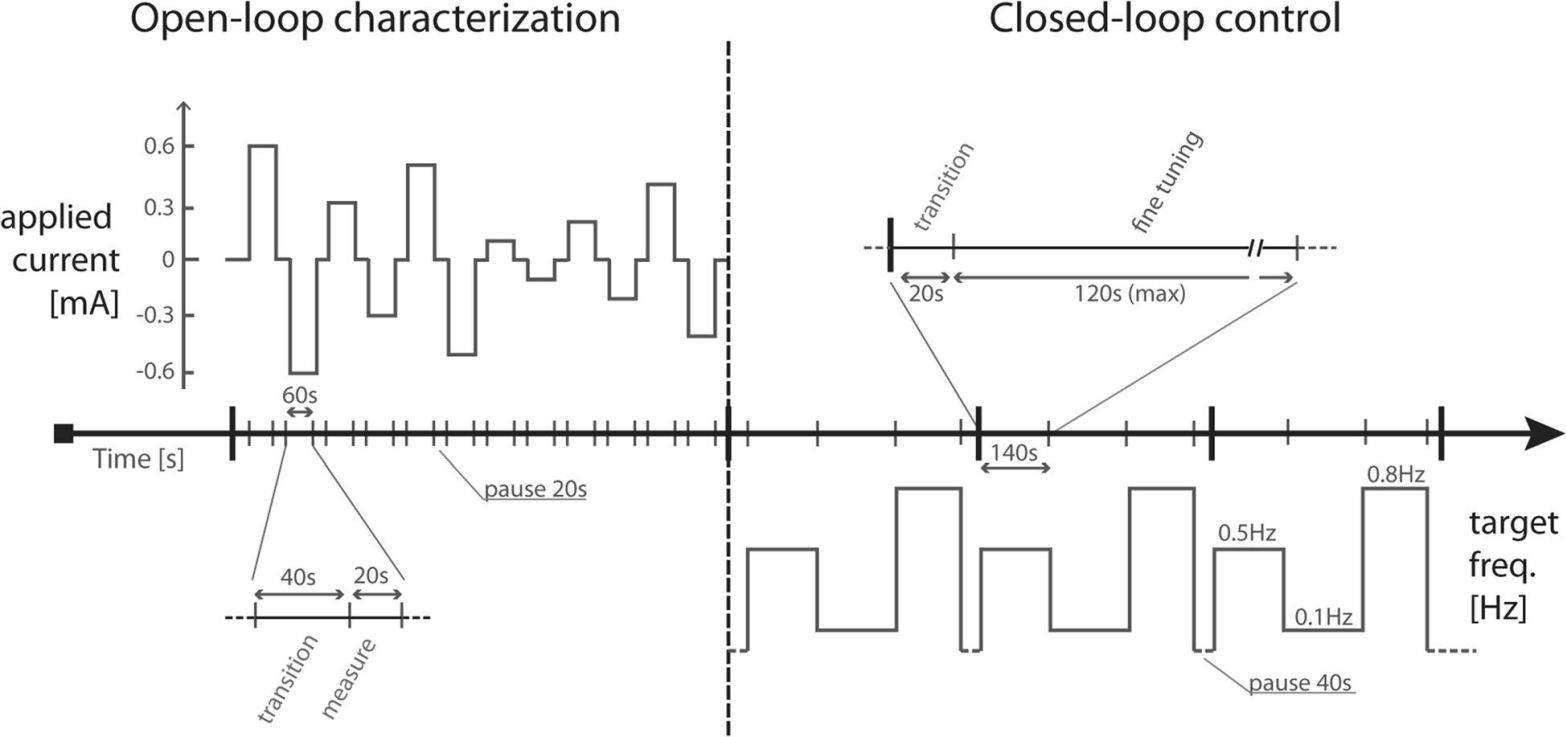
Experimental protocol. Left, initial open-loop characterization part: top left, profile of the applied current. Stimulation phases last 60 s. Current levels span the [− 0.6 + 0.6] mA range with 0.1-mA steps. There were 20 s between successive stimulation phases. Right, closed-loop part: bottom right, profile of the desired target SO frequency. A stimulation pause of 40 s was enforced after every set of three targets (0.5, 0.1 and 0.8 Hz)

The experimental protocol consisted of two main parts (Fig. 4): an open-loop part, for response characterization of the cortical network response, and a closed-loop part, where the biological activity was steered towards the desired SO frequency.

During the open-loop characterization (Fig. 4, left) we stimulated the slice with a series of six different randomized current levels in the range [0.1 0.65] mA, injecting for each level first positive current and then negative current, and measuring the corresponding obtained SO frequency. Each current level was maintained for 60 s and in between the stimulation periods we introduced pauses of 20 s. For the entire open-loop characterization, we fixed the duration of the window for SO frequency evaluation at 20 s; hence, to measure the activity we waited 40 s after the current update. Data obtained from this phase were collected in a current-vs-frequency graph (Fig. 2c), which characterized the slice being tested. For each stimulation current, we averaged all the measured values of the slow oscillation frequency obtained during the stimulation interval at that specific intensity (58 values). Every slice responded differently to the stimulation (see inset in Fig. 2c), as previously demonstrated [28]. Duration of Up and Down states in the spontaneous activity determined reactivity of the network to the applied electric fields.

In the second part of the protocol we activated the closed-loop control: it autonomously adjusted the current to steer the slice activity towards the desired targets. We chose three target values: 0.5, 0.1 and 0.8 Hz. For each slice we repeated three times the sequence of the three targets (Fig. 4, right). The duration of the measuring window was adjusted according to the target values such that, on average, ten Up states would be included in every measure. We also imposed a maximum limit of 40 s for the window duration. Thus, when the target was 0.8 Hz the measuring windows lasted 12.5 s, for 0.5 Hz we used a 20 s window and for 0.1 Hz a 40 s one. The closed-loop took advantage of the open-loop characterization made specifically for each slice by using, as a first guess for the current, a value obtained from the characterization data. As in the open-loop characterization, there was a “transition” phase before the start of data acquisition soon after the first estimated current was applied. After the transition phase, a fine-tuning closed-loop phase took place. The maximum duration of such a phase was set to 120 s. In case the target was reached, the system tried to maintain it for the following 40 s before passing on to the next target value.

During the initial experimental trials we tested 15 slices using different versions of the protocol, observing trends of the SO frequency modulation and optimizing the system and the experimental protocol. Although we faced some experimental issues, those initial tests provided valuable results that we discuss in the next section. After the test and troubleshooting phase was over, we applied the final stimulation protocol on 5 slices. The results presented in the following session refer to these 5 cases.

## Results

### Reaching the target frequencies

Cortical slices spontaneously engaged in the generation of the typical pattern of slow (< 1 Hz) oscillatory activity that has been proposed to be the default emergent activity of the cortical network [13, 14]. In a previous study [28] we demonstrated that the frequency of SO could be modulated in vitro by means of DC electric fields. In particular, positive (depolarizing) fields increased the SO frequency, while negative (hyperpolarizing) fields decreased it. Such modulation was proportional to the intensity of electric fields on a logarithmic scale. We aimed then to construct a closed-loop system (Fig. 1) able to steer the SO frequency toward specific targets, by stimulating the slices with DC current, as a proof of concept that the frequency of cortical SO can be controlled online at will.

Throughout the run, the closed-loop system tuned the current amplitude to induce the desired slice activity. In Fig. 2a we report 3 s of recording obtained with a fixed stimulation intensity of 0.3 mA. In this case the SO frequency of the slice being tested was about 1 Hz. Up states lasted 0.22 s on average (range [0.19 0.28] s, variance 0.0019 s), and their duration did not vary with the stimulation amplitude [28]. On the contrary, the duration of the Down states was finely modulated by the electric fields, decreasing for positive fields and increasing for negative fields. Therefore, in this example, the duration of Down states decreased by raising the stimulation level. For this particular slice the mean SO frequency in the absence of stimulation was 0.44 Hz.

To validate our closed-loop system we tested the final protocol (Fig. 4) in 5 cortical slices. Previous results [28] demonstrated that the intensity of the cortical response to the DC stimulation varies across slices. Thus, for each slice, an open-loop characterization was performed through the application of DC stimulation at randomized intensities for periods of 60 s. The recorded responses were rapidly fitted (Fig. 2c) in order to be able to make a proper guess on the stimulation intensity to be applied for each of the 3 closed-loop targets that we imposed (0.5 Hz, 0.1 Hz and 0.8 Hz). In all cases the closed-loop DC stimulation succeeded to steer the SO frequency to the desired target.

**Fig. 5:**
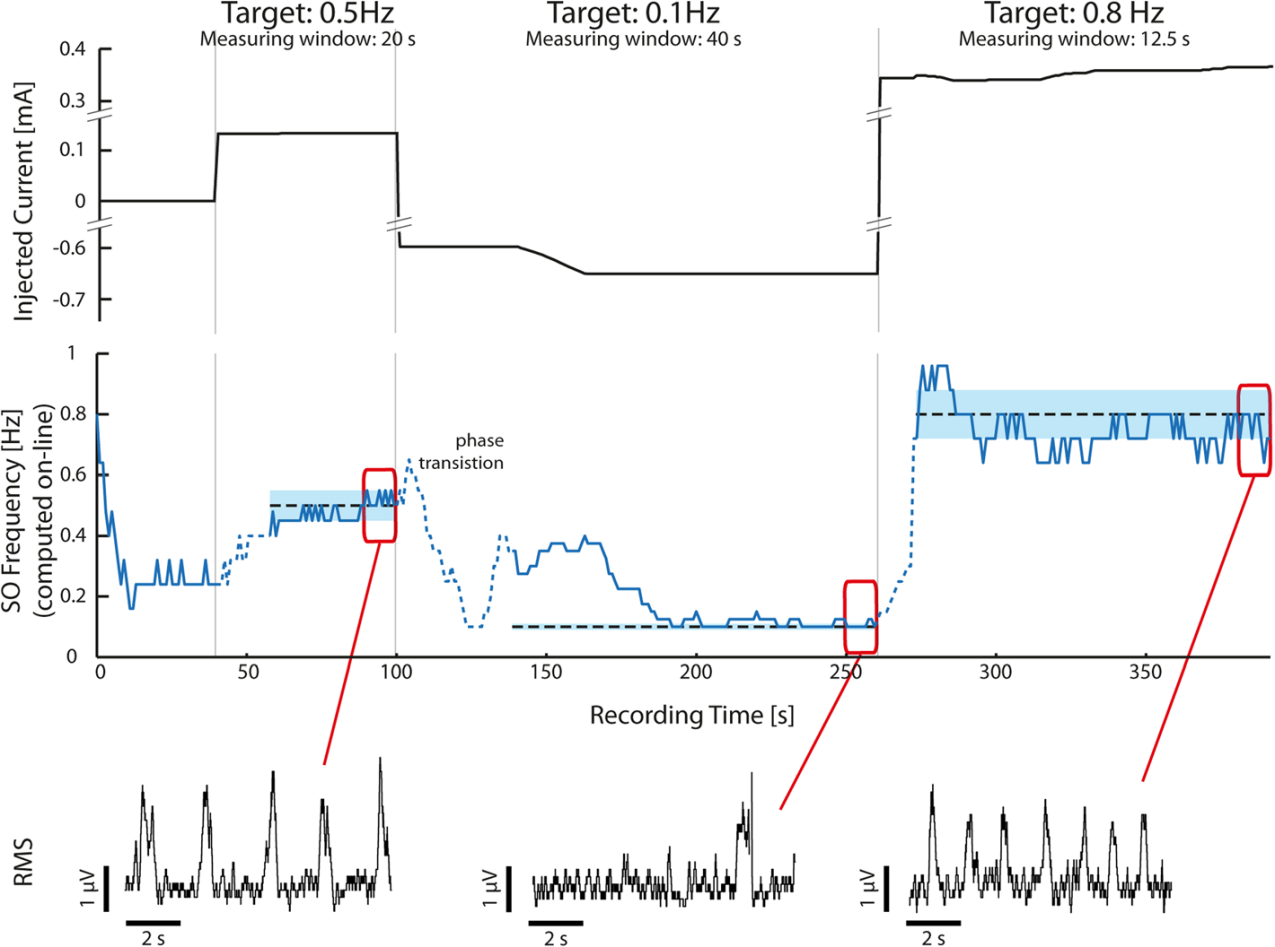
A closed-loop run. Recording (400 s) from the closed-loop part of the protocol. Top panel: black trace represents the applied current. Central panel: blue trace represents the online measure of the SO frequency. The line is dashed during the transition phase. The fine-tuning closed-loop phases start soon after the transition phase. Dashed black lines represent the desired target frequency levels (0.5, 0.1 and 0.8 Hz). Around the target frequency, the blue shade represents the tolerance range, that was set at 10% of the target frequency (0.01 Hz, 0.05 Hz and 0.08 Hz respectively). MUA traces are shown for the last 10 s of each target phase. Bottom panel: three sections of the recording illustrating the expanded areas indicated. Each section illustrates one of the reached oscillatory frequencies. SO, slow oscillations; RMS, voltage root mean square to identify occurrence of SO

Figure 5 reports data from one of the runs in the closed-loop phase. The SO frequency was modulated towards three successive target frequencies (0.5 Hz, 0.1 Hz and 0.8 Hz). For every target frequency, we started with an initial current-guess derived from the open-loop characterization phase. Then, after a transition-phase as long as the measuring window set for that target (see Methods), a fine tuning was started to bring and keep the SO frequency inside the tolerance range around the target. For the first target (0.5 Hz), the system made an estimation of the necessary current injection that was right and a short-lasting fine-tuning phase was necessary. For the second target (0.1 Hz), the initial guess for current injection was not enough to reach the target, and the network went into a frequency of oscillation of ca. 0.38 Hz. Then, the closed-loop acted to progressively reduce the stimulation current, achieving a stable frequency of oscillation around 0.1 Hz. For the third target (0.8 Hz), the current kept increasing trying to compensate for a too-low SO frequency. In this last case, even though we did not reach a final stable equilibrium, we kept the slice activity close to the desired target. The closed-loop continuously measured the activity and fine-tuned the stimulation, thus allowing both for the correction of imprecise current guesses and for slice activity stabilization over long time periods. We also report the MUA traces during the last 10 s of every phase (Fig. 5, bottom). We remark here that the duration of the window used to measure the SO frequency varied accordingly to the target frequency: it was set to 20 s when the target was 0.5 Hz, to 40 s for 0.1 Hz and 12.5 s for 0.8 Hz (see *Methods* for details).

### Flexibility of the system and consistency of the network response

**Fig. 6:**
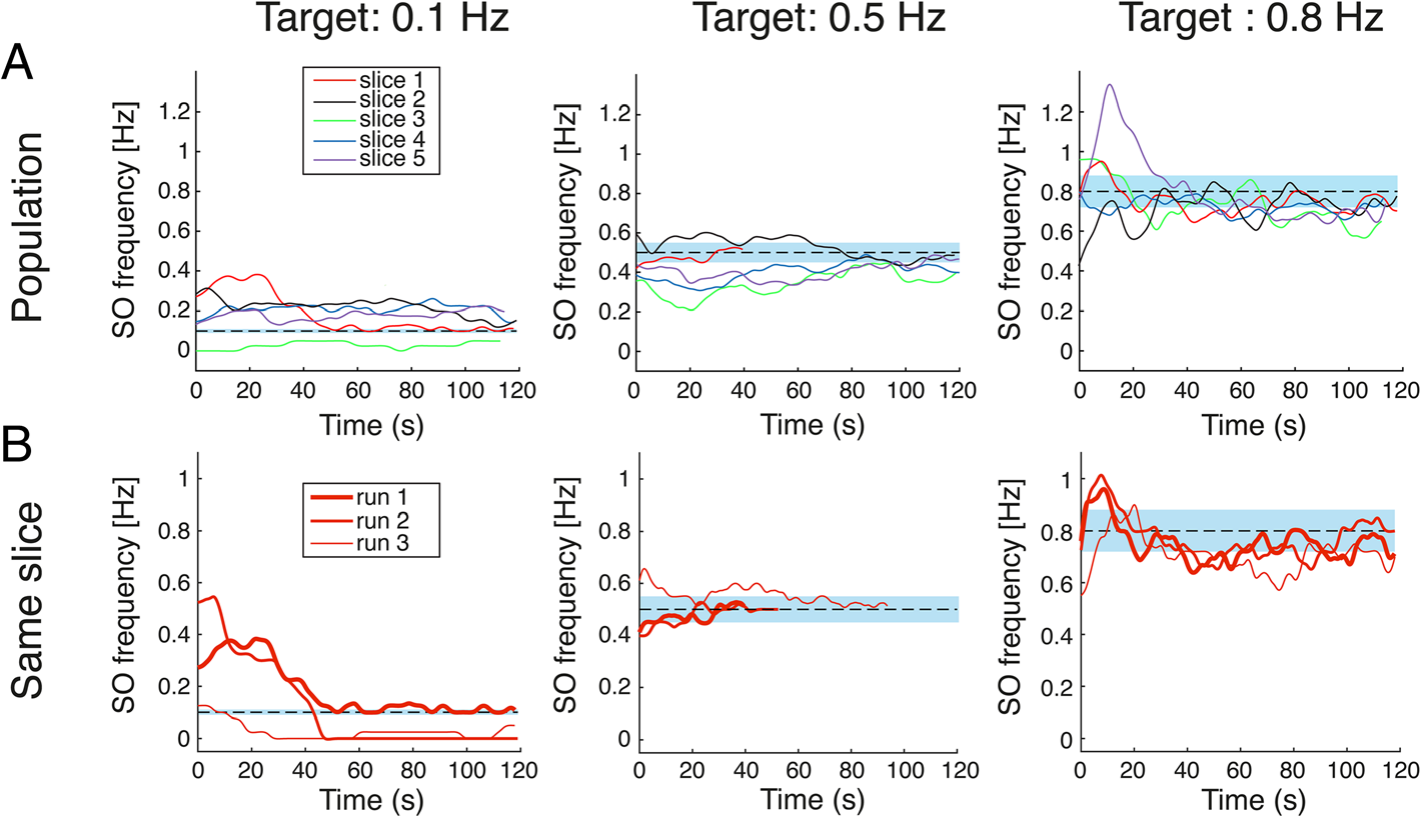
System flexibility and stability. **a** Behavior of SO frequency for all the slices (*n* = 5, color-coded). At *t* = 0, the closed-loop fine-tuning phase starts. In some cases (particularly at target 0.1 Hz), the frequency did not reach the target in the maximum time imposed by our protocol (120 s). The slope of the patterns, particularly for target 0.5 Hz and 0.8 Hz, suggests that the frequency would have reached the target over time. The same cannot be said for what concerns the 0.1 Hz, where many times the reached frequency was strongly decreased to a value between 0 and 0.2 Hz. **b** Data acquired from repetitions of one slice (slice 1) during a single uninterrupted series of 3 closed-loop protocols complete runs. Differences between the repetitions of the same target-reaching phase exist: the closed loop compensates for such unpredictable variations. Shadow bars represent the tolerance range, set at 10% of the target frequency (0.01 Hz, 0.05 Hz and 0.08 Hz respectively)

As mentioned before, different slices responded with different intensity to DC stimulation, in a manner strongly dependent on the initial activity [28]. DC fields act on the activity produced by the local network during the Down state, increasing or decreasing the firing rate (with depolarizing or hyperpolarizing fields, respectively) and changing the probability of the production of the following Up state [28]. Networks with high initial excitability (higher SO frequency) have small room for modulation at the intensities that we tested, thus hardly showing a strong response to the stimulation [28]. The closed-loop is an ideal setup to keep networks with such a heterogeneous behavior under control (Fig. 6a). In order to make the plots easier to read, the frequency patterns were smoothed by a cubic spline (Matlab ‘*csaps*’ function) with a smoothing parameter of 0.5, resampled at 100 Hz, and color-coded to illustrate the response of the different slices. Only the fine-tuning phase is illustrated in Fig. 6.

Independently from the departing frequency, the combination of current guessing and fine-tuning allowed the system to bring the oscillations close to the desired frequency. For the target 0.1 Hz, across all slices the mean SO frequency in the last 10 s of each run was 0.19 ± 0.1 Hz (mean ± SD), thus with an average deviation from the target of 0.09 Hz. For the targets 0.5 Hz and 0.8 Hz, the average SO frequency in the last 10 s was respectively 0.43 ± 0.06 Hz (deviation from target 0.07 Hz) and 0.75 ± 0.04 (deviation from target 0.05 Hz). In some cases, the frequency did not reach the target in the maximum time imposed by our protocol (120 s). The slope of the patterns, particularly for the targets 0.5 Hz and 0.8 Hz, suggests that the frequency would have reached the target over time. The same cannot be said for the 0.1 Hz target, where many times the reached frequency was strongly decreased to a value between 0 and 0.2 Hz but, within the time and current amplitude constrains that we imposed, it instead remained constant without reaching exactly 0.1 Hz. The 120 s limit derives from the experimental need not to “stress” the slice for too long to avoid deterioration of the network, and from the electrochemical degradation of the stimulation electrodes when high currents are applied for very long periods. When comparing the slice response among reiterations of the protocol, we often observed differences among repetitions of the same target-reaching phase (Fig. 6b). Differences are evident both in the departing points and in the time-evolution of the oscillatory patterns. In the illustrative case in Fig. 6b, when the target was 0.1 Hz (left column) the slices responded similarly to the first current guess twice (run 1 and run 2), while the following evolution of the SO pattern was different across the two cases (target reached only in the first runs). In the third run, in contrast, the slice response was completely different even at the first stimulation guess. For the 0.5 Hz target, the response was short and very similar twice (run 1 and run 2), while during the third run a longer fine-tuning phase was needed to reach the target. Finally, three very similar responses were observed when the target was 0.8 Hz, as probably there was little room for activity variations at such oscillation frequency, when the applied current was so high. These heterogeneities in the response could probably be related to some fatigue effects, to the intrinsic variability of the slice activity, or to some other environmental effects such as electrode oxidation or some unavoidable slight changes in temperature or solution flow. Nevertheless, the closed-loop system was able to steer the activity towards the desired target. Among all the recordings, when the target was set to 0.1 Hz, the activity often stabilized at a lower level (Fig. 6): at such low frequencies, a much longer time period would probably be necessary to evaluate whether the observed behavior was actually an incorrect stable equilibrium or a long-lasting undershoot.

### System stability

**Fig. 7:**
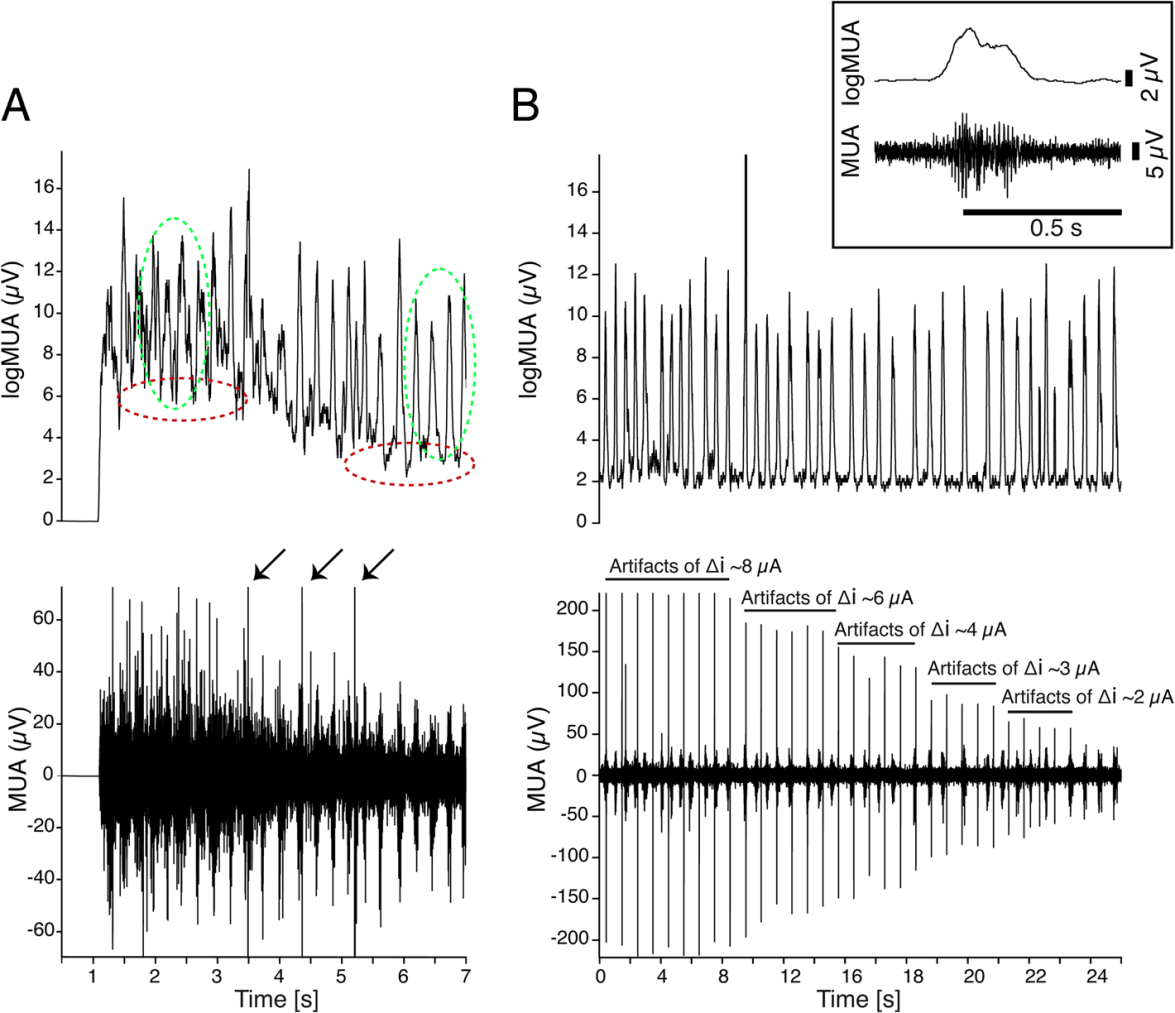
Artifact management and adaptive behavior to large current steps. **a** Top panel: firing rate (logMUA), first 7 s of recording after a large (~ 1 mA) step in the injected current (from ~ − 0.4 mA to ~ 0.6 mA). An adaptation phenomenon is visible. The red and green dashed circles highlight respectively the changes in the MUA baseline and in the amplitude of the Up states during and after the adaptation phase. Bottom panel: the corresponding MUA trace. The arrows mark three artifacts due to three small (< 50 μA) current updates. **b** Top panel: firing rate (logMUA) illustrating slow oscillations. Bottom panel: spikes in the MUA trace are artifacts due to current updates: the amplitude of the artifacts is proportional to the current jump. Artifacts are removed before computing the MUA trace (top panel) and do not affect the Up state detection. MUA, multiunit activity. Inset, one expanded Up state. Top, log MUA. Bottom, MUA

To obtain the control level described above, the first issue we encountered in this system involved artifacts in the recordings due to the current intensity update: every change in the current applied to the slices created an artifact in the recordings consisting of oscillations in the LFP signal baseline that caused the saturation of the acquisition system for several seconds. Our custom stimulator was designed paying particular attention to avoid the generation of undesired artifacts whose amplitude was reduced with respect to those observed in the same setup and preparation with commercial stimulators. With the custom stimulator, this kind of artifact was reduced so that the artifacts appeared as small spikes in the MUA traces (Fig. 7a and b, bottom), with the amplitude depending on the current step (Fig. 7b, bottom) while the duration was short enough to be able to be automatically distinguished from the Up states, which lasted at least 100 ms (Fig. 7b, top inset). Furthermore, thanks to the digital trigger sent back to the recording system, we were able to know precisely the moment in which the current was changed. This allowed us to remove online the majority of the artifacts from the filtered MUA traces (Fig. 7a and b, top). We note here that the commonly used artifact removal techniques consisting in disconnecting the pre-amplifiers for a short time-period around the stimulation is not useful in our context where we never detach the stimulation but instead keep adjusting its level.

Interestingly, the short artifacts generated by the current injection seemed to have no effect on the emerging activity of the network, either when they occurred during Down states or during Up states. We did not observe any network responses to the artifacts, as they just appeared in the recording trace and were mainly due to the non-infinite CMRR (common-mode-rejection-ratio) of the differential pre-amplifier. We also noted that the Up and Down states amplitude in the frequency range considered for the MUA traces remained unchanged across different amplitudes of artifacts (Fig. 7b, top).

Since the artifact amplitudes were proportional to the stimulation change, when the current step was large enough there was still a short period in which the signal went out of scale (see Fig. 7a for a representative case). Stimulation aligned at *t* = 0, and activity returned to a measurable range at around *t* = 1.5 s, much earlier than with other commercial stimulators. Since the signal did not go out of scale for long, we were able to observe that the network needed a few seconds of adaptation to return to its normal oscillatory activity (Fig. 7a). The example in Fig. 7a shows the first 7 s of a stimulation phase in which the current jump was about 0.1 mA (from ~ − 0.4 mA to ~ 0.6 mA). During this short period, the baseline of the MUA traces was increased (as highlighted by the red dashed circles in Fig. 7a) while the amplitude of the Up states remained almost unchanged (as highlighted by the green dashed circles in Fig. 7a). This effect was, to our knowledge, not an artifact in the recordings, but a response of the network activity to a large variation in the DC field.

## Discussion

In this study we designed, tested and demonstrated a closed-loop system that can control the frequency of SO generated by the cerebral cortex by means of DC current stimulation. It is a proof of concept that this rhythmic activity that emerges from cortical networks can be regulated within some limits, a principle that has both research and medical applications. To succeed, we solved several challenges in different fields ranging from artifact-related issues to real-time data analysis, to problems purely related to experiment execution. We discuss these interesting challenges in the following paragraphs.

### Challenges faced by a closed-loop system that are derived from the physiology

Our designed system falls into the category of semi-automatic systems, meaning that it can work alone but it also allows for user intervention online. This possibility to automatically and manually intervene provides the flexibility of our system, which also responds to the natural variability of the network oscillatory activity. We tested our system on the SO spontaneously generated by the cortical network [14], that consist of Up states (active periods of neuronal firing) and Down states (relatively silent periods). Automatic identification of Up states can be difficult due to many factors, ranging from the mechanisms of the network response to the changes in SO frequency caused by an applied electric field through DC stimulation. Changes in the SO frequency due to DC stimulation are related to an increase in the activity levels at high frequencies (200–1500 Hz), which corresponds to neuronal spikes, in the Down states [28]. Depolarizing fields induce an increase in excitability, and thus in the firing rate during Down states. The increase of firing rate during the Down states enhances the probability of triggering new Up states, thus resulting in a higher SO frequency. But it also results in a more challenging separation between Up and Down states, since there is more activity now in the Down states, and therefore a more difficult online autonomous identification of the Up states (see Methods section), in particular when injected currents were close to the maximum (around 0.6 mA). In most cases, the method for automatically setting the identification thresholds in the MUA traces worked fine; in a few cases (usually when injecting 0.5–0.6 mA), however, user intervention to change the Up state-identification threshold on the fly was needed, which was done without any interruption in the protocol.

### Challenges derived from the continuous DC stimulation and its updates

Particularly challenging was the managing of the artifacts introduced in the recordings by the jumps in the level of injected currents, which in the first versions of the system looked almost as large as Up states. By reducing the noise produced by the hardware during current updates and by adopting software solutions (see above), we were able to reduce the appearance of such artifacts to short-lasting spikes with amplitudes proportional to the current jump (Fig. 7b). We could then easily avoid the artifacts in the identification of Up states by setting a threshold on their minimum duration.

A significant reduction of the out-scaling of the recording in response to large steps in the current level was also achieved (Fig. 7a). This illustrates that the system initially reaches a certain frequency and then slowly adapts a stationary state-frequency for a given electric field intensity. Since the spontaneously emergent rhythmic patterns are the result of both synaptic (excitation, inhibition) and intrinsic properties (ionic channels, membrane properties) of the network’s neurons, every new level of network excitability needs a period to settle into a new balance of the different mechanisms resulting in a stable oscillatory frequency.

Prolonged application of high DC currents, without alternation of polarization, induces certain degradation of the stimulation electrodes by electrochemical reaction of the AgCl with the ACSF bath, consequently affecting the homogeneity of the applied fields [28]. Prolonged application of high currents could eventually also damage the tissue. Unfortunately, both the amplitude and the duration of the applied currents were defined in real-time by the closed-loop and are not predictable a priori. We just imposed some hard limits and this, in some cases, prevented the frequency from reaching its target (Fig. 6) In any case, permitted current ranges were sufficiently extended to allow for a proper modulation of the activity of most slices. Limits can be easily expanded to user wishes, and degradation of the electrodes could be solved by using a different material: gold or platinum are good candidates. We used silver-silver chloride (Ag/AgCl) to be consistent with previous studies from ourselves and others.

### Improving the control

A set of possible tools ranging from dedicated hardware to real-time operating systems could be adopted to reduce latencies in the loop and to access faster dynamics (for a review see [7]). In our case, to gain further control over the SO dynamics, apart from reducing latencies it would also be interesting to acquire data simultaneously from many spatially distributed electrodes, to impose different electric fields to different areas of the tissue and to explore more complex and dynamically adjustable stimulation patterns. This would allow us to control and understand the mechanisms underlying the SO dynamics in a more detailed way.

The intensity of the response of in-vitro networks to DC stimulation varies along with the initial spontaneous activity. In particular, the intensity correlates with Up and Down state durations in control conditions [28]. In some slices it is more difficult to achieve modulation of the SO frequency, especially for “extreme” targets such as 0.1 Hz or 0.8 Hz. In those cases, the distribution of the logarithm of the frequency across current intensities looks more like a sigmoid rather than a linear distribution, and this makes it almost impossible to reach boundaries frequencies, at least with the limits that we imposed on timing and current intensities.

### Potential applications

The modulation of SO frequency represents a starting point for a more complete closed-loop control of cortical activity. Once the identification of activity phases is achieved (in this case Up and Down states), it becomes easier to compute other activity parameters besides Up state frequency. It will then be possible to switch the system to control any of these activity parameters, as long as there is a specific model describing the system’s changes in relation to the DC stimulation, and as long as the timing of the changing parameters fits with the computation latencies.

The interest in closed-loop systems is corroborated by recent tests of closed-loop systems in humans. An implantable closed-loop system is thought to ameliorate the conditions of patients affected by Parkinson’s disease or suffering from epileptic crises (for a review see [42]). Because the imbalance between excitation and inhibition that leads to an alteration of the network activation patterns could be the basis of neurological or neurodegenerative diseases, such as Parkinson’s disease [24] and Alzheimer’s disease [43, 44], implantation of closed-loop systems may aid these clinical populations too. In fact, tDCS has been shown to have good potential for a wide range of clinical applications [45–47] (for a recent review see [48]).

Regarding SO, it is now rather established that enhancement of SO during slow wave (NREM) sleep can be a useful approach to enhance cognition at all ages (for a recent review see [17]). In particular, SO during sleep enhanced through transcranial magnetic stimulation have been found to enhance memory [49]. There is also memory enhancement while activated during wakefulness [50], although through the activation of higher frequencies. Furthermore, slow waves have been entrained through auditory close-loop stimulation, also enhancing memory [51].

Our system is a proof of concept of the possibility of regulating SO by means of direct current in a closed-loop way, a principle that here we demonstrate in an in-vitro section of the cerebral cortex, but it can be applied in vivo. The possibility of an autonomous system that controls and sets SO during patient sleep could be an interesting application for future developments of our system. The closed-loop would ensure a controlled application of low intensity currents in order to re-adjust SO parameters in case they get aberrant during the NREM sleep phases of patients, leading to a more focused use of this treatment. In this perspective, future improvements of the system would involve the control of many parameters simultaneously, an objective that will pose challenges on the reduction of timing and delays of the computation system.

## Conclusions

We designed a closed-loop system that can control the frequency of cortical slow oscillations. The designed system was able to identify Up states in real-time, measure their frequency, and autonomously adjust the intensity of the injected current such that a target frequency set by the user would be reached. To minimize the time required to hit the target frequency, an initial current guess was obtained from an exponential model of the network’s reaction to DC electric fields [28]. By finely and continuously tuning the injected current to keep the frequency inside a tolerance range around the target frequency, the system compensated for natural and unpredictable fluctuations arising from the spontaneous activity of the network. Our system paves the way for future studies on in-vitro and in-vivo oscillatory dynamics of the cerebral cortex.

## Abbreviations

AC: Alternating current
ACSF: Artificial cerebrospinal fluid
DC: Direct current
LFP: Local field potential
MUA: Multiunit activity
SO: Slow oscillations
tDCS: Transcranial direct current stimulation
